# Frailty in relation to general and oral health-related quality of life among older adults in municipal acute care

**DOI:** 10.1016/j.tjfa.2026.100207

**Published:** 2026-10-17

**Authors:** Leonor Roa Santervas, Eva Skovlund, Ingrid Beate Ringstad, Espen Saxhaug Kristoffersen, Katrine Gahre Fjeld, Lene Hystad Hove, Janicke Liaaen Jensen, Torgeir Bruun Wyller, Rita Romskaug

**Affiliations:** a Institute of Clinical Medicine, University of Oslo, Oslo, Norway; b Department of Geriatric Medicine, Oslo University Hospital, Oslo, Norway; c City of Oslo Health Agency, Municipality of Oslo, Norway; d Department of Public Health and Nursing, The Norwegian University of Science and Technology, Trondheim, Norway; e Department of Oral Surgery and Oral Medicine, University of Oslo, Oslo, Norway; f Department of General Practice, Institute of Health and Society, University of Oslo, Norway; g Department of Neurology, Akershus University Hospital, Lørenskog, Norway; h Department of Cariology and Gerodontology, University of Oslo, Oslo, Norway

**Keywords:** Frailty, Health-related quality of life, Oral health-related quality of life, Geriatrics, Municipal in-patient acute care unit

## Abstract

Frailty is associated with poorer health-related quality of life (HRQoL) in older adults, but studies rarely assess general and oral HRQoL concurrently in acute care populations. Objectives: To examine the associations between frailty and general as well as oral HRQoL among older adults admitted to a municipal in-patient acute care (MipAC) unit. Design: Cross-sectional study. Setting: Oslo MipAC unit, Norway. Participants: A total of 382 patients aged 70 years or older admitted to the MipAC unit between January and September 2023. Measurements: Frailty was assessed using the Clinical Frailty Scale (CFS). General HRQoL was measured with the 15D instrument and a single-item global life satisfaction question. Oral HRQoL was assessed using the Oral Health Impact Profile-14 (OHIP 14). Associations between frailty and HRQoL were analyzed using linear, ordinal logistic, and binary logistic regression. Exploratory analyses of 15D and OHIP-14 dimensions were performed using non-parametric trend tests. Results: Increasing frailty was significantly associated with lower 15D scores (adjusted B -0.053, 95% CI -0.061 to -0.045), higher odds of lower life satisfaction (adjusted OR 1.19, 95% CI 1.02 to 1.39), and higher odds of reporting oral health impacts on the OHIP-14 (adjusted OR 1.25, 95% CI 1.05 to 1.48). Most 15D dimensions declined with increasing frailty, except distress. For OHIP-14, significant associations were observed for functional limitation, physical pain, and psychological disability. Conclusions: Frailty assessed with the CFS was consistently associated with reduced general and oral HRQoL in older adults admitted to a MipAC unit. These findings support the clinical relevance of simple frailty assessment during acute admission and suggest that it may help identify patients at risk of poorer quality of life.

## Highlights

• Frailty assessed with the CFS was associated with poorer general and oral HRQoL in older adults admitted to a MipAC unit.
• Each one-point increase in CFS was associated with lower 15D scores and higher odds of reduced life satisfaction.
• Increasing frailty was associated with higher odds of reporting oral health impacts on the OHIP-14.
• Most 15D domains declined with increasing frailty.
  Simple frailty assessment during acute admission may help identify older patients at risk of poorer quality of life.

## Graphical abstract

**Figure:**
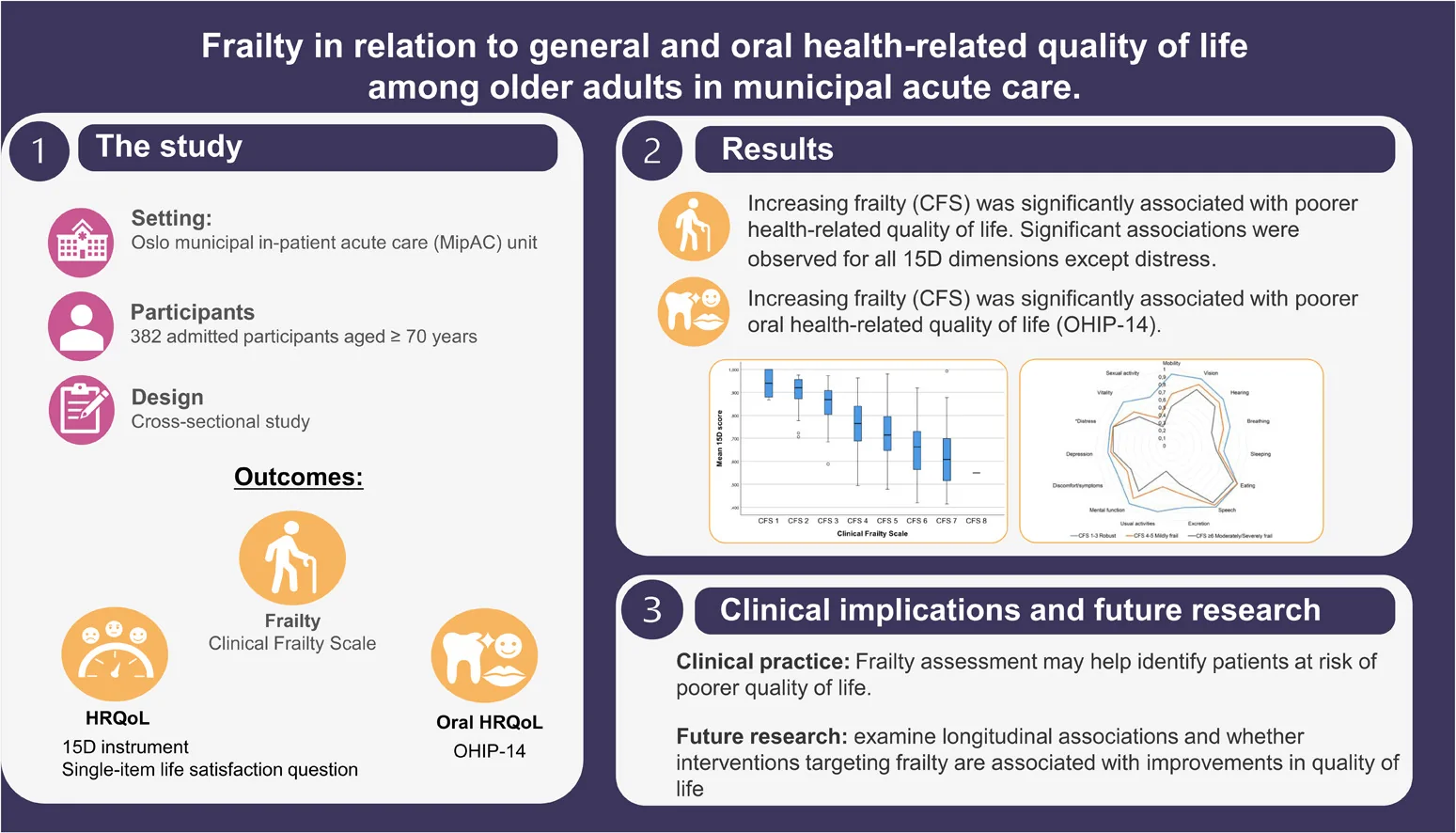

## Introduction

Health-related quality of life (HRQoL) captures aspects of health that extend beyond traditional biomedical indicators and is particularly relevant in older adults, in whom conventional clinical metrics may not fully capture the lived experience [[1], [2], [3]]. By encompassing subjective physical, psychological, and social dimensions of functioning, HRQoL provides important information about the burden of illness and disability, and may support clinical decision making, resource allocation, and person-centered care [4].

Older adults are especially vulnerable to impaired HRQoL due to the cumulative burden of chronic conditions, functional limitations, and increasing dependence. Frailty, defined as a clinical syndrome of diminished physiological reserve and increased vulnerability to stressors, is particularly relevant in this context and is a strong predictor of morbidity, hospitalization, and mortality [5]. Several studies have also shown that increasing frailty is associated with poorer HRQoL [[6], [7], [8], [9]].

Although this association is well established, important gaps remain. Many previous studies have relied on generic measures that provide only a global HRQoL score, and few have examined how frailty relates to specific HRQoL domains. Fewer still have combined multidimensional HRQoL instruments with simple and clinically applicable frailty measures like the Clinical Frailty Scale (CFS) [10]. Oral health is another important, but often overlooked, dimension of quality of life in older adults [11,12]. Emerging evidence indicates that both poor oral health and reduced oral HRQoL are associated with frailty, but general and oral HRQoL are rarely assessed concurrently in the same study population, and this limits our understanding of how frailty relates to different but complementary aspects of quality of life [[13], [14], [15], [16], [17], [18]]. The use of validated multidomain instruments such as the 15D instrument (15D) [19] and the Oral Health Impact Profile-14 (OHIP-14) [20], alongside a global life satisfaction measure, may provide a more nuanced understanding of the association between frailty and quality of life.

The aim of this study was therefore to explore the association between frailty, assessed with the CFS, and three complementary quality of life measures in older adults: general HRQoL assessed by the 15D instrument, overall life satisfaction assessed by a single-item question, and oral HRQoL assessed by the OHIP-14. In addition, we examined the individual dimensions of the 15D and OHIP-14 to explore whether some domains appeared more strongly associated with frailty than others.

## Methods

### Study design and participants

The study was performed at the Oslo municipal in-patient acute care (MipAC) unit, with participant enrollment between January and September 2023. Patients admitted to the MipAC unit must have been evaluated by a physician at primary care, an emergency clinic, or at the hospital before admission to confirm medical stability and well-defined diagnoses. To be eligible for the study, participants had to be 70 years or older, not isolated for infection control purposes, and able to communicate in Norwegian. Exclusions applied to those with a life expectancy of less than one month, or if staff members at the MipAC unit (nurses or physicians) deemed participation unfeasible due to clinical conditions such as drowsiness, delirium, or acute psychiatric episodes. As it was impossible to foresee every scenario in which inclusion would be ethically or practically inappropriate, these conditions were not predetermined. Patients anticipated to be discharged within 24 hours, and those already enrolled, were also excluded. More details regarding recruitment and the study population have previously been published [21,22].

### Data collection

The study team consisted of two study nurses and two physicians responsible for enrolling patients, managing and compiling medical and demographic data, and conducting overall health evaluations. Information was collected directly from the participants, their medical records, and from relatives when necessary. For individuals who declined participation in the study, age and sex were recorded.

### Measures

#### Frailty

Frailty was assessed using the CFS, a nine-point clinical judgment scale ranging from 1 (very fit) to 9 (terminally ill) [10]. Scores were assigned by one of the study nurses or study physicians, all of whom had received training in CFS assessment. The rating reflected the patient’s habitual functional abilities, cognition, and level of dependence 2 weeks preceding the acute illness that led to admission [23]. The recommended classification tree was consulted for guidance when needed [23], and uncertain ratings were discussed within the study team and resolved by consensus. As patients with an expected survival of less than one month were excluded, CFS 9 was not applicable. Analyses were conducted using both the continuous CFS scale and aggregated categories: 1 to 3 (robust), 4 to 5 (mildly frail), and ≥6 (moderately to severely frail) [24,25]. This categorization allowed both statistical modeling and clinically meaningful stratification.

#### General health-related quality of life

General HRQoL was assessed with two complementary instruments: the 15D instrument and a single-item global life satisfaction question. These instruments were chosen to capture distinct aspects of general quality of life; the former focuses primarily on self-perceived health-related functioning and symptoms, whereas the latter captures overall subjective well-being.

The 15D instrument evaluates 15 dimensions: mobility, vision, hearing, breathing, sleeping, eating, speech, excretion, usual activities, mental function, discomfort/symptoms, depression, distress, vitality, and sexual activity [19,26]. The full 15D questionnaire is provided in the Supplementary Material. Each dimension is rated on a five-point ordinal scale. The instrument provides both a profile of individual dimension scores and a single index score of overall HRQoL. The index score is calculated using population-based utility weights and ranges from 0 (poorest HRQoL) to 1 (excellent HRQoL) [26,27]. For cross-sectional comparisons, a difference of 0.015 in the 15D index score is considered the minimum clinically important difference [27]. In addition to analyzing the overall 15D score, we examined each of the 15 dimensions to explore whether the associations with frailty differed across domains.

Overall life satisfaction was assessed with the single-item question: “Thinking about your life at the moment, would you say that you by and large are satisfied with life, or are you mostly dissatisfied?” Responses were given on a seven-point Likert scale ranging from 1 (very satisfied) to 7 (very dissatisfied) and were collapsed into three categories: 1–3 (very satisfied to somewhat satisfied), 4 (neutral), and 5–7 (somewhat dissatisfied to very dissatisfied). This item has been used in previous Norwegian studies and was included to capture a broad global appraisal of subjective well-being [[28], [29], [30], [31]]. Single-item life satisfaction measures have shown acceptable validity in large population-based studies [32,33].

For both measures, participants were instructed to report their habitual condition 2 weeks prior to the acute illness that led to admission. Both the 15D questionnaire and the single-item life satisfaction question were interviewer-administered.

#### Oral health-related quality of life

Oral HRQoL was assessed using the OHIP-14, a questionnaire comprising 14 items across seven dimensions: functional limitation, physical pain, psychological discomfort, physical disability, psychological disability, social disability, and handicap [20,34]. The first three dimensions primarily reflect the experience of oral health problems, while the remaining dimensions reflect their consequences for daily functioning and social participation.

Respondents report the frequency of oral health-related problems experienced during the past year using a five-point scale that ranges from 0 (“never”) to 4 (“very often”), generating a sum score from 0 to 56 where a higher score indicates poorer oral HRQoL [20]. In addition to analyzing the sum score, we explored each of the seven dimensions to investigate whether specific domains of oral HRQoL were more or less strongly associated with frailty. Due to a pronounced left-skewed distribution of OHIP-14 responses in our sample, both the sum score and the individual dimension scores were dichotomized for analysis: 0 (indicating no impact) versus > 0 (indicating some level of impact) [35].

### Data analysis and statistics

Descriptive statistics are presented as means with standard deviations (SD) for continuous variables and as counts with percentages for categorical variables. Group differences were analyzed using parametric or non-parametric tests, as appropriate.

For the 15D, up to three missing items were imputed according to the developers' guidelines [19,26]. For OHIP-14, no imputation was performed; participants with missing items were excluded from analyses of the total sum score. However, in the exploratory analyses of OHIP-14 dimensions, participants were included provided that all items within the relevant dimension were completed.

Unadjusted and adjusted regression analyses were conducted to examine the association between frailty (CFS) and the three quality-of-life outcomes. Linear regression was used for the 15D index score, with results reported as unstandardized regression coefficients (B) and 95% confidence intervals (CI). Ordinal logistic regression was used for the single-item life satisfaction measure, and binary logistic regression was used for the dichotomized OHIP-14 outcome. Results from these models are reported as odds ratios (OR) and 95% CI.

To estimate the association between frailty and each outcome while reducing confounding, the same prespecified covariates were included in all adjusted models: age, sex, education, and number of regular medications at admission. These variables were selected a priori based on clinical relevance in geriatric populations and to ensure comparability across the three outcome models. The purpose of the adjustment was to obtain a more robust estimate of the association between frailty and the outcomes, rather than to evaluate the independent effects of the covariates themselves. The number of regular medications was chosen instead of the Charlson Comorbidity Index (CCI) as a pragmatic indicator of treated chronic disease burden, as medication count may capture chronic conditions not included in the CCI.

Regression diagnostics included visual inspection of residual distributions, assessment of multicollinearity, and evaluation of linearity assumptions in the logistic models. No major violations of model assumptions were identified. Statistical significance was defined as p< 0.05.

In exploratory sub-analyses, the individual dimensions of the 15D were examined using the Jonckheere–Terpstra test to assess trends across CFS categories. For the OHIP-14 dimensions, dichotomized scores (0 vs >0) were analyzed across CFS categories using the chi-square test for trend.

Data were analyzed using IBM SPSS Statistics for Windows version 29.0 (IBM Corp). Figures were created using IBM SPSS Statistics and Microsoft Excel.

## Results

Throughout the recruitment period, 1277 patients aged ≥70 years were screened, of whom 382 were included in the study. Reasons for exclusion are given in Supplementary Figure 1. No differences in age or sex were observed between participants and those who declined participation (Supplementary Table 1). Table 1 presents characteristics of the study population. The mean (SD) age of participants was 84 (7), and 72% were women. Admission reasons are presented in Supplementary Figure 2.

**Table 1:** Patient characteristics (N = 382).

Characteristic	Value
Demographic and social characteristics	
Age, mean (SD), years	84 (7)
Female sex, n (%)	275 (72)
Living alone, n (%)	294 (77)
Receiving home nursing care, n (%)	193 (51)
Higher education, n (%) (n = 366)	133 (36)
Clinical characteristics	
Charlson Comorbidity Index (not adjusted for age) (0–33), mean (SD), score	1.6 (1.8)
Regular medications in use at admission, mean (SD), number	6.7 (3.5)
Frailty	
Clinical Frailty Scale (1–8), mean (SD), score	4.6 (1.5)
Clinical Frailty Scale 1–3, n (%)	98 (26)
Clinical Frailty Scale 4–5, n (%)	166 (44)
Clinical Frailty Scale ≥6, n (%)	118 (31)
Quality of life	
15D (0–1), mean (SD), single index score (n = 359)	0.75 (0.14)
Single-item question (1–7), mean (SD), score (n = 353)	3.45 (1.49)
Single-item question categories, n (%) (n = 353)	
1–3: Very satisfied/satisfied/somewhat satisfied	181 (51.3)
4: Neither satisfied nor dissatisfied	124 (35.1)
5–7: Somewhat dissatisfied/dissatisfied/very dissatisfied	48 (13.6)
OHIP-14 (0–56), mean (SD), score (n = 340)	3.84 (6.74)
OHIP-14 sum score >0, n (%)	208 (61.2)

OHIP-14, Oral Health Impact Profile-14; SD, standard deviation.

Because questionnaire completion may be associated with patient characteristics, we compared participants who completed all three quality-of-life instruments with those who did not. Participants who did not complete all three instruments were older, more frail, more often lived alone, and more frequently received home nursing care. They also had a higher comorbidity burden, whereas sex, education, and number of regular medications were similar between the groups (Supplementary Table 2).

The unadjusted and adjusted associations between CFS score and the three quality-of-life outcomes are presented in Table 2.

**Table 2:** Associations between a one-point higher Clinical Frailty Scale score and quality-of-life outcomes.

**Outcome**	**Model**	**Unadjusted**		**Adjusted**	
		**n**	**Estimate (95% CI)**	**n**	**Estimate (95% CI)**
15D index score	Linear regression	359	B −0.060 (−0.067 to −0.053)	355	B −0.053 (−0.061 to −0.045)
Life satisfaction	Ordinal logistic regression	353	OR 1.31 (1.14 to 1.51)	350	OR 1.19 (1.02 to 1.39)
Any OHIP-14 impact	Binary logistic regression	340	OR 1.27 (1.10 to 1.48)	338	OR 1.25 (1.05 to 1.48)

B, unstandardized regression coefficient; CI, confidence interval; OHIP-14, Oral Health Impact Profile-14; OR, odds ratio.

Adjusted models included age, sex, education, and number of regular medications at admission. Life satisfaction was categorized as satisfied, neutral, or dissatisfied. Any OHIP-14 impact was defined as a total OHIP-14 score >0.

### Frailty and 15D

Increasing frailty was significantly associated with lower 15D scores, both in the unadjusted (B −0.060, 95% CI −0.067 to −0.053, n = 359) and the adjusted model (B −0.053, 95% CI −0.061 to −0.045, n = 355). The decline in 15D index score across CFS categories is illustrated in Fig. 1.

**Fig. 1:**
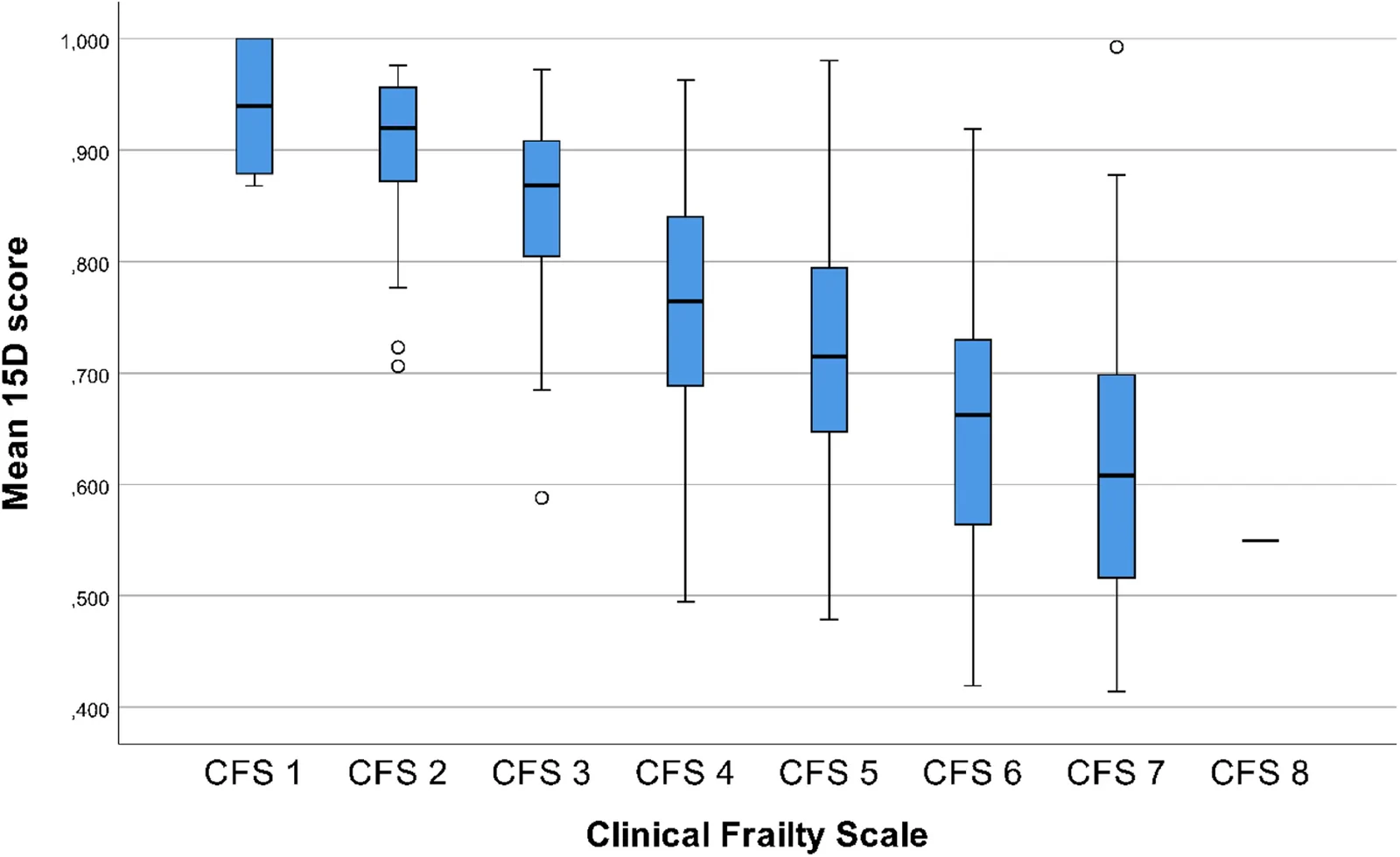
Mean 15D score across Clinical Frailty Scale (CFS) categories (n = 359).

Except for distress, all 15D dimensions showed a statistically significant trend of declining scores with increasing frailty (Fig. 2 and Supplementary Table 3).

**Fig. 2:**
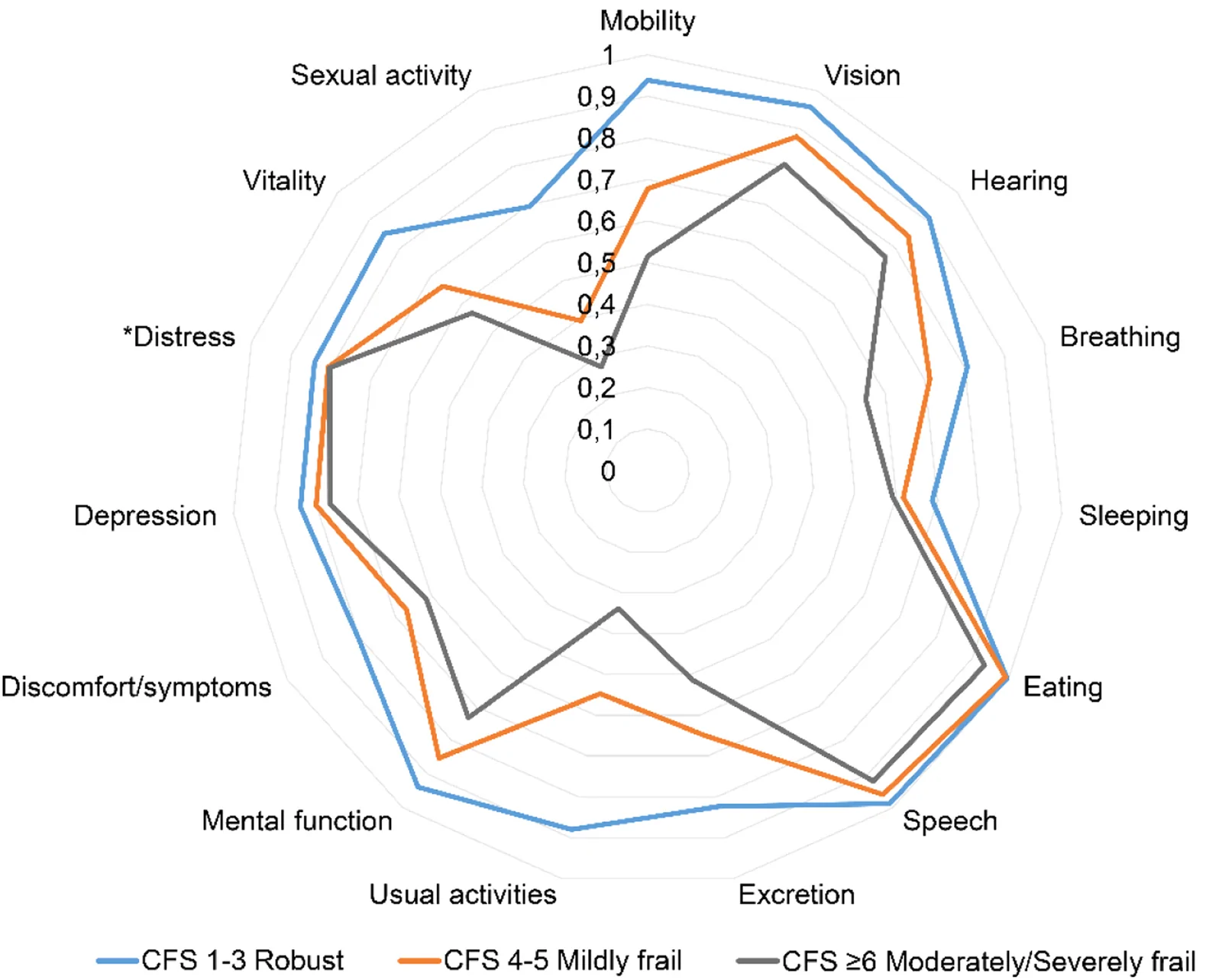
Mean 15D dimension scores across aggregated Clinical Frailty Scale (CFS) categories. CFS, Clinical Frailty Scale *p > 0.05 Mean scores of the 15D dimensions are displayed for three aggregated CFS categories (1–3, 4–5, ≥6). As the categories are discrete, the connecting lines do not indicate continuity but are included to facilitate visual comparison across groups.

### Frailty and the single-item question

Frailty was also associated with poorer life satisfaction. The odds of reporting a category of lower satisfaction increased by 31% per unit increase in CFS in the unadjusted model (OR 1.31, 95% CI 1.14 to 1.51, n = 353) and by 19% in the adjusted model (OR 1.19, 95% CI 1.02 to 1.39, n = 350).

To further explore this relationship, we compared patients with moderate to severe frailty (CFS ≥6) who reported high life satisfaction (single-item score 1–3) with those reporting lower satisfaction (single-item score 4–7). There was no clear difference between the two groups in age, sex, comorbidity, support, or 15D scores (Supplementary Table 4).

### Frailty and OHIP-14

Frailty was also significantly associated with impaired oral HRQoL. Patients with higher CFS scores had greater odds of reporting oral health-related impacts on the OHIP-14, with OR 1.27 (95% CI 1.10 to 1.48, n = 340) per unit increase in CFS in the unadjusted model and OR 1.25 (95% CI 1.05 to 1.48, n = 338) in the adjusted model.

At the dimension level, significant trends across CFS categories were observed for functional limitation, physical pain, and psychological disability (Fig. 3 and Supplementary Table 5), while other domains showed numerical but non-significant differences.

**Fig. 3:**
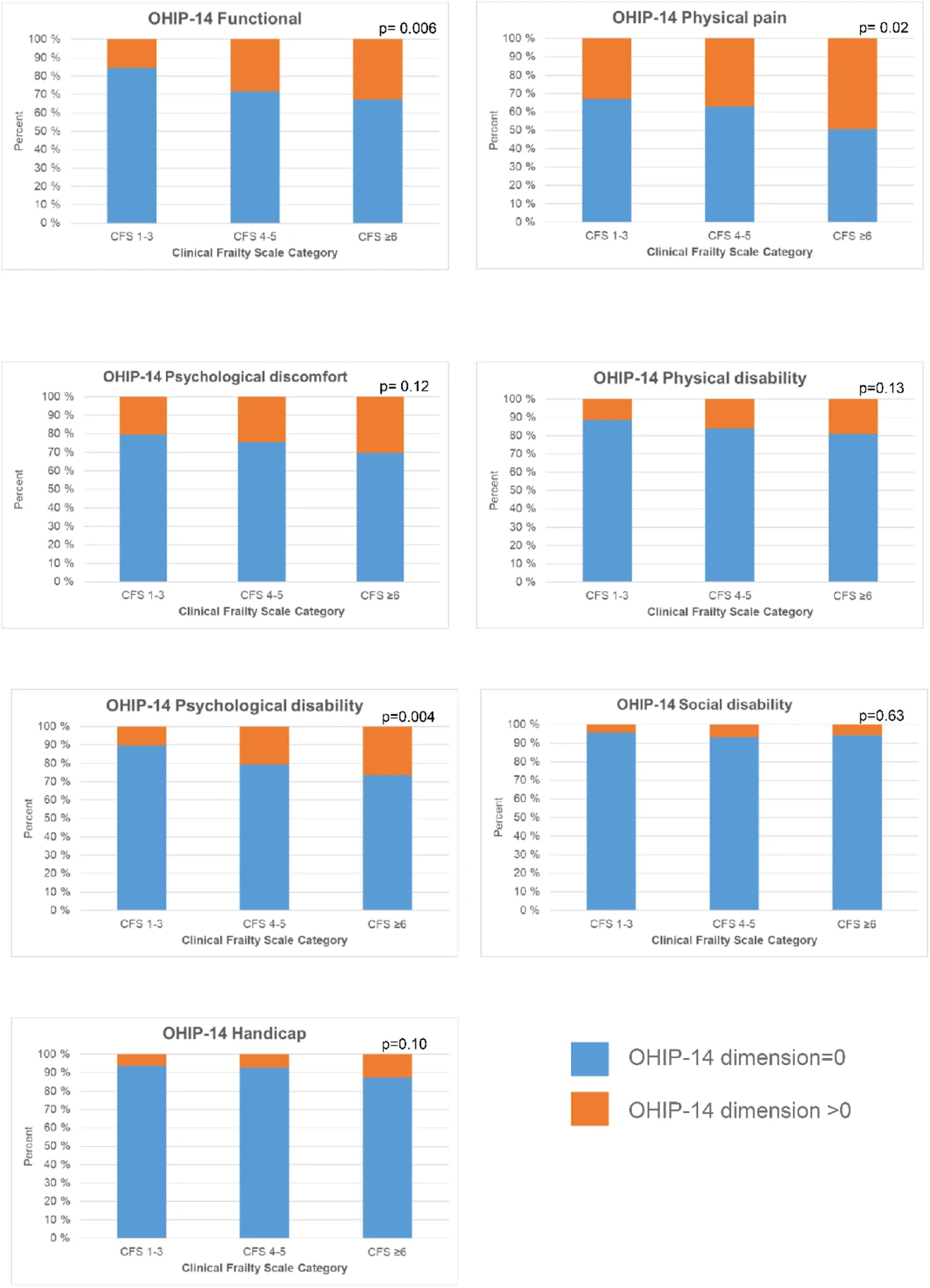
Distribution of scores (0 and >0) across different OHIP-14 dimensions by CFS categories: CFS 1–3 (robust), CFS 4–5 (mildly frail) and CFS ≥ 6 (moderately/severely frail). CFS, Clinical Frailty Scale; OHIP-14, Oral Health Impact Profile-14.

## Discussion

The high proportion of participants with CFS scores ≥4 and the frequent use of home nursing services reflect the considerable vulnerability and care needs of this municipal acute-care population. In this study, frailty assessed with the CFS was associated with both general and oral HRQoL. Increasing frailty was associated with lower scores on the 15D instrument, lower life satisfaction, and a higher likelihood of reporting oral health-related impacts on the OHIP-14. These associations were evident across most HRQoL dimensions, highlighting the broad and multidimensional relationship between frailty and quality of life in this population.

### General HRQoL

Frailty was strongly associated with HRQoL in both the 15D and the single-item life satisfaction measure. In the adjusted analysis, a one-point higher CFS score was associated with a 0.053-point lower 15D index score, a difference greater than the suggested minimum clinically important difference for cross-sectional comparisons [27]. Functional dimensions of the 15D, such as mobility and the ability to perform usual activities, showed the strongest associations with frailty. This is not unexpected, given that the CFS itself is largely based on functional status and that many 15D dimensions reflect physical capacity. Nonetheless, the association extended beyond functional aspects: most psychological and symptom-related dimensions, apart from distress, also declined with increasing frailty. These findings are consistent with previous studies showing that frailty is associated with poorer psychological well-being, including symptoms of depression [8,9,[36], [37], [38]]. This pattern may partly reflect the close interrelationship between functional capacity, mood, vitality, and symptom burden, but also suggests that frailty is linked to broader aspects of well-being that cannot be fully explained by functional impairment alone.

The single-item life satisfaction measure provided a complementary perspective. Despite its broad and subjective nature, it showed a clear gradient across frailty levels, supporting its value as an overall indicator of well-being in this setting. Single-item approaches have been shown to correlate well with established multi-item scales, both in older adults and in large national panel studies [32,39,40]. However, such measures do not identify which specific aspects of health or life circumstances account for lower satisfaction, and responses may also be influenced by cultural norms, reporting tendencies, and processes of psychological adaptation. For example, some individuals may hesitate to report dissatisfaction, while others may maintain relatively high life satisfaction despite functional decline through adaptation, resilience, or continued emphasis on broader sources of meaning such as social connectedness, autonomy, and existential well-being [41]. This may partly explain why 38% of participants with high frailty nevertheless reported high life satisfaction. In our exploratory comparison, these patients did not show clear differences in 15D score, sex, living situation, or support compared with those with the same degree of frailty who reported lower life satisfaction.

### Oral HRQoL

Our study highlights that frailty is also associated with impaired oral HRQoL. Although most patients reported none or only very low levels of oral health-related impacts on the OHIP-14, frailty was associated with higher odds of experiencing at least *some* negative impact. Significant associations were particularly evident in the OHIP-14 domains of functional limitation, physical pain, and psychological disability. These findings should be interpreted in light of prior evidence suggesting a bidirectional relationship: frailty may worsen oral health status and shape how older adults perceive their oral health, while poor oral health may in turn contribute to frailty through pathways such as impaired nutrition and systemic inflammation [13,17,42]. The absence of consistent associations across all OHIP-14 dimensions may reflect the overall low prevalence of oral health-related issues in this study, but could also indicate that certain items, such as embarrassment or social disadvantage, may be perceived as less relevant in the context of acute somatic illness and advanced frailty [20,35,43]. In this setting, oral health-related problems not directly related to pain or basic function may have been underreported or less salient in patients’ recall.

### Limitations and strengths

Our study has several limitations. The cross-sectional design precludes causal inference. In addition, although participants were asked to report their habitual status prior to the acute illness leading to admission, data collection took place during an acute care stay. This approach was chosen to align the quality of life assessments with the CFS, which is intended to reflect premorbid frailty. Nevertheless, the reported quality of life may have been influenced not only by recall bias, but also by symptoms, stress, fatigue, or other aspects of the acute illness, meaning that these measures may not fully represent premorbid status. The exclusion of patients isolated for infection control purposes, as well as those deemed unable to participate due to clinical reasons, may have introduced selection bias and may have led to underestimation of the full burden of frailty in this setting. Furthermore, patients who did not complete all three quality-of-life instruments were more frail and had a higher comorbidity burden than those who completed all three. Their underrepresentation may have attenuated the observed associations, resulting in an underestimation of their magnitude in the broader MipAC population. Given the left-skewed distribution of OHIP-14 scores, the scores were dichotomized for analysis. This resulted in loss of information and may have reduced the sensitivity to detect more subtle gradients in symptom burden. Finally, although the adjusted models included key demographic and health-related covariates, oral health variables were not accounted for. This may have affected the findings, especially those related to oral HRQoL, although the use of the same covariate set across analyses improved comparability between outcomes.

This study also has several strengths. It includes a large and clinically relevant sample of acutely ill older adults and is, to our knowledge, the first to employ the CFS to assess associations with both the 15D and the OHIP-14. The use of a multidimensional general HRQoL instrument, a global life satisfaction measure, and an oral HRQoL instrument within the same study provided complementary perspectives on quality of life. Analyses were conducted both at the summary score level and across individual dimensions, allowing a more nuanced understanding of which aspects of HRQoL are affected by frailty. Furthermore, data collection was conducted by trained personnel using structured procedures, enhancing internal validity.

### Clinical implications

Our findings support the clinical relevance of frailty assessment in routine care, including in municipal acute care and other non-geriatric settings where such evaluations are not yet common practice. The CFS is a simple and feasible tool, applicable in acute contexts, and its association with both general and oral HRQoL suggests that it may help identify patients at risk of reduced quality of life. The finding that some highly frail participants nevertheless reported high life satisfaction may reflect adaptation and resilience and underscores the importance of considering patients’ own perspectives and goals. Early recognition of frailty may enable clinicians to anticipate functional decline, tailor interventions to support independence, and guide care planning. The observed associations with oral HRQoL further suggest that oral health should be considered an integral part of care for older adults in acute settings.

### Future directions

Future research should explore longitudinal trajectories of frailty and HRQoL, assess whether interventions targeting modifiable aspects of frailty are associated with improvements in general and oral HRQoL, and explore whether the present findings are generalizable to older adults in other care settings.

## Conclusions

Frailty assessed by the CFS was consistently associated with reduced general and oral HRQoL, with similar trends across most HRQoL dimensions. These findings support the clinical relevance of simple frailty assessments in older adults during acute admission. Frailty assessment may help identify patients at risk of poorer quality of life and may inform a more comprehensive assessment of patient needs and care planning.

## Abbreviations

15D: 15D instrument

CCI: Charlson Comorbidity index

CFS: clinical frailty scale

CI: confidence interval

HRQoL: health-related quality of life

MipAC: municipal in-patient acute care

OHIP-14: oral health impact profile-14

OR: odds ratio

## Data availability

Data may be available upon reasonable request to the authors. Availability is dependent on approval from the Regional Ethics Committee for medical research in the South-East of Norway and the Data Protection Officer at Oslo University Hospital.

## Declaration of generative Ai and Ai-assisted technologies in the manuscript preparation process

During the preparation of this work the authors used GPT-4 Omni provided by the University of Oslo solely to improve readability and language. After using this tool, the authors carefully reviewed and edited the content as needed and take full responsibility for the content of the published article.

## Funding

Our research received funding from the South-Eastern Norway Regional Health Authorities (grant number 2,022,066). The design, management, analysis and reporting of the study were carried out without any influence from the funding body.

## Ethics approval

The study was carried out in accordance with the Declaration of Helsinki and approved by the Regional Committee for Medical and Health Research Ethics of South-East Norway (REK 477,258) and the Data Protection Officer at Oslo University Hospital.

## Consent to participate

All participants provided written, informed consent. If patients were unable to provide consent themselves, consent from their next of kin was sought.

## CRediT authorship contribution statement

**Leonor Roa Santervas:** Writing – review & editing, Writing – original draft, Visualization, Methodology, Investigation, Formal analysis, Data curation, Conceptualization. **Eva Skovlund:** Writing – review & editing, Visualization, Supervision, Methodology, Formal analysis, Conceptualization. **Ingrid Beate Ringstad:** Writing – review & editing, Methodology. **Espen Saxhaug Kristoffersen:** Writing – review & editing, Supervision, Methodology, Conceptualization. **Katrine Gahre Fjeld:** Writing – review & editing, Methodology. **Lene Hystad Hove:** Writing – review & editing, Methodology. **Janicke Liaaen Jensen:** Writing – review & editing, Methodology. **Torgeir Bruun Wyller:** Writing – review & editing, Supervision, Methodology, Funding acquisition, Conceptualization. **Rita Romskaug:** Writing – review & editing, Writing – original draft, Supervision, Resources, Project administration, Methodology, Investigation, Funding acquisition, Data curation, Conceptualization.

## Declaration of competing interest

The authors declare that they have no known competing financial interests or personal relationships that could have appeared to influence the work reported in this paper.
