# Supplementary material for: Frailty in relation to general and oral health-related quality of life among older adults in municipal acute care
Source: J Frailty Aging. 2026 Oct 17;15(5):100207. doi: 10.1016/j.tjfa.2026.100207 (PMC13695251; doi:10.1016/j.tjfa.2026.100207)
Supplement: Supplementary file 1 [file mmc1.docx]

## **SUPPLEMENTARY MATERIAL**

## Frailty in relation to general and oral health-related quality of life among older adults in municipal acute care

**Supplementary Figure 1.** Flow of participants through screening and questionnaire completion

* Additionally, 12 patients provided partial OHIP-14 data and were included in dimension-specific exploratory analyses

**Supplementary Table 1.** Comparison of age and sex between included patients and those who declined participation

| **Characteristic** | **Included patients** (n=382) | **Declined participation** (n=122) |
| --- | --- | --- |
| **Age,** mean (SD), years | 84 (7) | 86 (8) |
| **Sex,** female, n (%) | 275 (72) | 91 (75) |

**Supplementary Figure 2.** Admission reasons

If there were several reasons for admission, the most relevant was the one recorded.

**Supplementary Table 2.** Comparison of characteristics between participants who completed all three quality-of-life instruments and those who did not complete all three instruments

| **Characteristic** | **Completed all three instruments**  **(n=335)** | **Did not complete all three instruments**  **(n=47)** | **Between-group difference**  **(95% CI)** |
| --- | --- | --- | --- |
| **Demographic and social characteristics** |  |  |  |
| Age, mean (SD), years | 84 (7) | 86 (6) | -2 (-4 to 1)* |
| Female, n (%) | 245 (73) | 30 (64) | 0.09 (-0.05 to 0.24)** |
| Living alone, n (%) | 252 (75) | 42 (89) | -0.14 (-0.24 to -0.04)** |
| Receiving home nursing care, n (%) | 155 (46) | 38 (81) | -0.35 (-0.47 to -0.22)** |
| Higher education, n (%) | 122 (37)*** | 11 (33)**** | 0.03 (-0.14 to 0.20)** |
| **Clinical characteristics** |  |  |  |
| Charlson Comorbidity Index (not adjusted for age) (0-33), mean (SD), score | 1.5 (1.7) | 2.2 (2.3) | 0 (-1 to 0)* |
| Regular medications in use at admission, mean (SD), number | 6.7 (3.6) | 6.8 (3.3) | 0 (-1 to 1)* |
| Clinical Frailty Scale (1-8), mean (SD), score | 4.5 (1.5) | 5.4 (1.2) | -1 (-1 to 0)* |

CI, confidence interval; SD, standard deviation

* Median difference (95% CI); Hodges-Lehmann

** Difference in proportions (95% CI); asymptotic, no continuity correction

*** n=333

**** n=33

**Supplementary Table 3.** 15D dimension scores across aggregated CFS categories (n=359).

|  | **CFS 1-3**  Mean (SD) | **CFS 4-5**  Mean (SD) | **CFS ≥6**  Mean (SD) | **p value*** |
| --- | --- | --- | --- | --- |
| **01. Mobility** | 0.94 (0.13) | 0.68 (0.20) | 0.52 (0.15) | <.001 |
| **02. Vision** | 0.96 (0.12) | 0.88 (0.20) | 0.81 (0.28) | <.001 |
| **03. Hearing** | 0.91 (0.16) | 0.84 (0.21) | 0.77 (0.25) | <.001 |
| **04. Breathing** | 0.81 (0.21) | 0.71 (0.28) | 0.55 (0.30) | <.001 |
| **05. Sleeping** | 0.69 (0.30) | 0.62 (0.32) | 0.59 (0.32) | 0.037 |
| **06. Eating** | 0.99 (0.04) | 0.99 (0.08) | 0.93 (0.16) | <.001 |
| **07. Speech** | 0.99 (0.07) | 0.96 (0.12) | 0.92 (0.17) | <.001 |
| **08. Excretion** | 0.82 (0.24) | 0.65 (0.30) | 0.51 (0.30) | <.001 |
| **09. Usual activities** | 0.88 (0.17) | 0.55 (0.27) | 0.34 (0.24) | <.001 |
| **10. Mental function** | 0.94 (0.14) | 0.85 (0.21) | 0.73 (0.29) | <.001 |
| **11. Discomfort/symptoms** | 0.80 (0.24) | 0.67 (0.33) | 0.61 (0.33) | <.001 |
| **12. Depression** | 0.84 (0.22) | 0.80 (0.23) | 0.77 (0.25) | 0.026 |
| **13. Distress** | 0.84 (0.23) | 0.81 (0.24) | 0.80 (0.25) | 0.289 |
| **14. Vitality** | 0.85 (0.23) | 0.66 (0.24) | 0.57 (0.28) | <.001 |
| **15. Sexual activity** | 0.70 (0.31) | 0.39 (0.26) | 0.27 (0.20) | <.001 |

CFS, Clinical Frailty Scale; SD, standard deviation

*Jonckheere-Terpstra test for trend

**Supplementary Table 4.** Characteristics of patients with CFS ≥6 according to single-item life satisfaction (satisfied [1–3] vs. neutral/dissatisfied [4–7]).

| **Characteristic** | **CFS ≥6** | |
| --- | --- | --- |
|  | **Single-item question** | |
|  | **1-3**  n=40 | **4-7**  n=65 |
| **Demographic and social characteristics** |  |  |
| Age, mean (SD), years | 86 (6) | 86 (7) |
| Female sex, n (%) | 27 (68) | 48 (74) |
| Living alone, n (%) | 32 (80) | 55 (85) |
| Receiving home nursing care, n (%) | 36 (90) | 62 (95) |
| Higher education, n (%) | 11 (28) | 15 (24) |
| **Clinical characteristics** |  |  |
| Charlson Comorbidity Index (0-33), mean (SD), score | 2.3 (2.1) | 2.3 (2.2) |
| Regular medications in use at admission, mean (SD), number | 7.8 (3.3) | 8.5 (4.1) |
| 15D (0-1), mean (SD), single index score | 0.67 (0.13) | 0.63 (0.12) |

SD, standard deviation

**Supplementary Table 5.** Associations between dichotomized OHIP-14 dimensions (0 vs >0) across aggregated CFS categories.

| **Dependent variable** | **CFS 1-3** | **CFS 4-5** | **CFS ≥6** | **p value*** |
| --- | --- | --- | --- | --- |
| **OHIP-14 dimension, category >0** | **n (%)** | **n (%)** | **n (%)** |  |
| Functional limitation (n=349) | 15 (15.5) | 42 (28.4) | 34 (32.7) | 0.006 |
| Physical pain (n=351) | 32 (33) | 55 (36.9) | 52 (49.5) | 0.02 |
| Psychological discomfort (n=351) | 20 (20.6) | 36 (24.3) | 32 (30.2) | 0.12 |
| Physical disability (n=350) | 11 (11.3) | 24 (16.1) | 20 (19.2) | 0.13 |
| Psychological disability (n=351) | 10 (10.3) | 31 (20.8) | 28 (26.7) | 0.004 |
| Social disability (n=349) | 4 (4.1) | 10 (6.8) | 6 (5.8) | 0.63 |
| Handicap (n=348) | 6 (6.2) | 11 (7.4) | 13 (12.6) | 0.10 |

CFS, Clinical Frailty Scale; OHIP-14, Oral Health Impact Profile-14

*Chi-square test for trend

**15D QUESTIONNAIRE (15D©)**

15D©/ Harri Sintonen ([www.15D-instrument.net](http://www.15D-instrument.net)). Reproduced with permission.

Please read through all the alternative responses to each question before placing a cross (x) against the alternative which best describes your present health status. Continue through all 15 questions in this manner, giving only one answer to each.

QUESTION 1. MOBILITY
1 ( ) I am able to walk normally (without difficulty) indoors, outdoors and on stairs.
2 ( ) I am able to walk without difficulty indoors, but outdoors and/or on stairs I have slight difficulties.
3 ( ) I am able to walk without help indoors (with or without an appliance), but outdoors and/or on stairs only with considerable difficulty or with help from others.
4 ( ) I am able to walk indoors only with help from others.
5 ( ) I am completely bed-ridden and unable to move about.

QUESTION 2. VISION
1 ( ) I see normally, i.e. I can read newspapers and TV text without difficulty (with or without glasses).
2 ( ) I can read papers and/or TV text with slight difficulty (with or without glasses).
3 ( ) I can read papers and/or TV text with considerable difficulty (with or without glasses).
4 ( ) I cannot read papers or TV text either with glasses or without, but I can see enough to walk about without guidance.
5 ( ) I cannot see enough to walk about without a guide, i.e. I am almost or completely blind.

QUESTION 3. HEARING
1 ( ) I can hear normally, i.e. normal speech (with or without a hearing aid).
2 ( ) I hear normal speech with a little difficulty.
3 ( ) I hear normal speech with considerable difficulty; in conversation I need voices to be louder than normal.
4 ( ) I hear even loud voices poorly; I am almost deaf.
5 ( ) I am completely deaf.

QUESTION 4. BREATHING
1 ( ) I am able to breathe normally, i.e. with no shortness of breath or other breathing difficulty.
2 ( ) I have shortness of breath during heavy work or sports, or when walking briskly on flat ground or slightly uphill.
3 ( ) I have shortness of breath when walking on flat ground at the same speed as others my age.
4 ( ) I get shortness of breath even after light activity, e.g. washing or dressing myself.
5 ( ) I have breathing difficulties almost all the time, even when resting.

QUESTION 5. SLEEPING
1 ( ) I am able to sleep normally, i.e. I have no problems with sleeping.
2 ( ) I have slight problems with sleeping, e.g. difficulty in falling asleep, or sometimes waking at night.
3 ( ) I have moderate problems with sleeping, e.g. disturbed sleep, or feeling I have not slept enough.
4 ( ) I have great problems with sleeping, e.g. having to use sleeping pills often or routinely, or usually waking at night and/or too early in the morning.
5 ( ) I suffer severe sleeplessness, e.g. sleep is almost impossible even with full use of sleeping pills, or staying awake most of the night.

QUESTION 6. EATING
1 ( ) I am able to eat normally, i.e. with no help from others.
2 ( ) I am able to eat by myself with minor difficulty (e.g. slowly, clumsily, shakily, or with special appliances).
3 ( ) I need some help from another person in eating.
4 ( ) I am unable to eat by myself at all, so I must be fed by another person.
5 ( ) I am unable to eat at all, so I am fed either by tube or intravenously.

QUESTION 7. SPEECH
1 ( ) I am able to speak normally, i.e. clearly, audibly and fluently.
2 ( ) I have slight speech difficulties, e.g. occasional fumbling for words, mumbling, or changes of pitch.
3 ( ) I can make myself understood, but my speech is e.g. disjointed, faltering, stuttering or stammering.
4 ( ) Most people have great difficulty understanding my speech.
5 ( ) I can only make myself understood by gestures.

QUESTION 8. EXCRETION
1 ( ) My bladder and bowel work normally and without problems.
2 ( ) I have slight problems with my bladder and/or bowel function, e.g. difficulties with urination, or loose or hard bowels.
3 ( ) I have marked problems with my bladder and/or bowel function, e.g. occasional 'accidents', or severe constipation or diarrhea.
4 ( ) I have serious problems with my bladder and/or bowel function, e.g. routine 'accidents', or need of catheterization or enemas.
5 ( ) I have no control over my bladder and/or bowel function.

QUESTION 9. USUAL ACTIVITIES
1 ( ) I am able to perform my usual activities (e.g. employment, studying, housework, freetime activities) without difficulty.
2 ( ) I am able to perform my usual activities slightly less effectively or with minor difficulty.
3 ( ) I am able to perform my usual activities much less effectively, with considerable difficulty, or not completely.
4 ( ) I can only manage a small proportion of my previously usual activities.
5 ( ) I am unable to manage any of my previously usual activities.

QUESTION 10. MENTAL FUNCTION
1 ( ) I am able to think clearly and logically, and my memory functions well
2 ( ) I have slight difficulties in thinking clearly and logically, or my memory sometimes fails me.
3 ( ) I have marked difficulties in thinking clearly and logically, or my memory is somewhat impaired.
4 ( ) I have great difficulties in thinking clearly and logically, or my memory is seriously impaired.
5 ( ) I am permanently confused and disoriented in place and time.

QUESTION 11. DISCOMFORT AND SYMPTOMS
1 ( ) I have no physical discomfort or symptoms, e.g. pain, ache, nausea, itching etc.
2 ( ) I have mild physical discomfort or symptoms, e.g. pain, ache, nausea, itching etc.
3 ( ) I have marked physical discomfort or symptoms, e.g. pain, ache, nausea, itching etc.
4 ( ) I have severe physical discomfort or symptoms, e.g. pain, ache, nausea, itching etc.
5 ( ) I have unbearable physical discomfort or symptoms, e.g. pain, ache, nausea, itching etc.

QUESTION 12. DEPRESSION
1 ( ) I do not feel at all sad, melancholic or depressed.
2 ( ) I feel slightly sad, melancholic or depressed.
3 ( ) I feel moderately sad, melancholic or depressed.
4 ( ) I feel very sad, melancholic or depressed.
5 ( ) I feel extremely sad, melancholic or depressed.

QUESTION 13. DISTRESS
1 ( ) I do not feel at all anxious, stressed or nervous.
2 ( ) I feel slightly anxious, stressed or nervous.
3 ( ) I feel moderately anxious, stressed or nervous.
4 ( ) I feel very anxious, stressed or nervous.
5 ( ) I feel extremely anxious, stressed or nervous.

QUESTION 14. VITALITY
1 ( ) I feel healthy and energetic.
2 ( ) I feel slightly weary, tired or feeble.
3 ( ) I feel moderately weary, tired or feeble.
4 ( ) I feel very weary, tired or feeble, almost exhausted.
5 ( ) I feel extremely weary, tired or feeble, totally exhausted.

QUESTION 15. SEXUAL ACTIVITY
1 ( ) My state of health has no adverse effect on my sexual activity.
2 ( ) My state of health has a slight effect on my sexual activity.
3 ( ) My state of health has a considerable effect on my sexual activity.
4 ( ) My state of health makes sexual activity almost impossible.
5 ( ) My state of health makes sexual activity impossible.

* Participants completed the validated Norwegian version of this instrument. The English-language version is provided for reference.
